# Evidence of Differential Effects of Vitamin D Receptor Variants on Epithelial Ovarian Cancer Risk by Predicted Vitamin D Status

**DOI:** 10.3389/fonc.2014.00286

**Published:** 2014-10-20

**Authors:** Jennifer Prescott, Kimberly A. Bertrand, Brett M. Reid, Jennifer Permuth-Wey, Immaculata De Vivo, Daniel W. Cramer, Kathryn L. Terry, Shelley S. Tworoger

**Affiliations:** ^1^Channing Division of Network Medicine, Department of Medicine, Brigham and Women’s Hospital and Harvard Medical School, Boston, MA, USA; ^2^Program in Genetic Epidemiology and Statistical Genetics, Department of Epidemiology, Harvard School of Public Health, Boston, MA, USA; ^3^Department of Epidemiology, Harvard School of Public Health, Boston, MA, USA; ^4^Department of Cancer Epidemiology, H. Lee Moffitt Cancer Center and Research Institute, Tampa, FL, USA; ^5^Obstetrics and Gynecology Epidemiology Center, Department of Obstetrics and Gynecology, Brigham and Women’s Hospital and Harvard Medical School, Boston, MA, USA

**Keywords:** ovarian cancer, vitamin D, polymorphism, haplotype, heterogeneity

## Abstract

**Introduction:** Experimental studies suggest vitamin D inhibits ovarian carcinogenesis. Yet, epidemiologic studies of ovarian cancer risk and lifestyle correlates of vitamin D status, plasma 25-hydroxyvitamin D [25(OH)D], or vitamin D receptor (*VDR*) variants have been inconsistent.

**Objective:** To evaluate *VDR* genetic associations by high vs. low predicted 25(OH)D, scores derived from known determinants of plasma 25(OH)D. To assess ovarian cancer associations with variants identified in genome-wide association studies (GWAS) of plasma 25(OH)D.

**Methods:** We genotyped up to seven *VDR* and eight 25(OH)D GWAS variants in the Nurses’ Health Studies (562 cases, 1,553 controls) and New England Case–Control study (1,821 cases, 1,870 controls). We estimated haplotype scores using expectation-maximization-based algorithms. We used unconditional logistic regression to calculate odds ratios (ORs) and 95% confidence intervals (CI). We combined study results using DerSimonian and Laird meta-analysis.

**Results:** Ovarian cancer risk increased per A allele of rs7975232 (*VDR*; OR = 1.12, 95% CI = 1.01–1.25) among all women. When stratified by predicted 25(OH)D, ovarian cancer was associated with rs731236 (*VDR*; per C allele OR = 1.31) and rs7975232 (OR = 1.38) among women with high predicted 25(OH)D, but not among women with low levels (*P* ≤ 0.009). We also observed heterogeneity by predicted 25(OH)D for the ovarian cancer association with *VDR* 3′ end haplotypes (*P* = 0.009). Of 25(OH)D-associated GWAS loci, rs7041 was associated with reduced ovarian cancer risk (per T allele OR = 0.92, 95% CI = 0.85-0.99), which did not differ by predicted 25(OH)D status.

**Conclusion:** Our study suggests an influence of *VDR* 3′ end variants on ovarian cancer risk may be observed in women with high predicted 25(OH)D, which remained even after taking multiple comparisons into consideration. Future studies are needed to confirm our results and explore further the relation between vitamin D exposure, genetic variants, and ovarian cancer risk.

## Introduction

Experimental studies suggest that vitamin D and its synthetic derivatives protect against ovarian carcinogenesis, exhibiting anti-proliferative, and pro-apoptotic effects in ovarian cancer cell lines (1–4) and antitumor activity in animal models (5, 6). The vitamin D receptor (VDR), which binds the biologically active form of vitamin D (1,25-dihydroxyvitamin D [1,25(OH)D]), is weakly to moderately expressed in normal ovarian cells and more strongly expressed in many ovarian cancer cells lines and tumor tissues (2, 7–11). Ecological studies generally support the link between vitamin D exposure and ovarian cancer risk, observing higher rates of ovarian cancer incidence and/or mortality among women living in more northern latitudes (12–16). However, observational studies using individual-level data on estimated UV radiation exposure, dietary and/or supplemental vitamin D intake, predicted 25(OH)D scores, and plasma 25(OH)D levels have been inconsistent (17–30).

Genetic studies may provide another line of evidence for the vitamin D pathway in ovarian carcinogenesis. To date, nine ovarian cancer genetic susceptibility loci have been identified by four genome-wide association studies (GWAS) (31–34). While known vitamin D pathway genes do not appear to reside at these loci, the newly established variants explain a relatively small proportion of excess familial risk (34). Additional common susceptibility loci are likely to exist, but will require alternate approaches such as evaluating gene–environment interaction to discover these associations. Common polymorphisms in the *VDR* are suspected to either directly affect or be in linkage disequilibrium (LD) with variants that influence vitamin D signaling (35), which would alter the biological response to vitamin D at the cellular level. Of the *VDR* variants that have been most extensively studied, the rs228570 T allele [a.k.a. *Fok*I restriction fragment length polymorphism (RFLP) “f” allele] creates a VDR protein that is three amino acids longer and less transcriptionally active than the protein product of the C allele (35). A recent meta-analysis including a total of 4,163 cases and 6,801 controls observed a significant 9% increase in ovarian cancer risk associated with each rs228570 T allele (36). While statistically significant associations with ovarian cancer risk were not observed in a meta-analysis for four other VDR variants, total sample sizes were smaller than that for rs228570, thus, reducing power to detect genetic associations (37). Additionally, vitamin D status of study participants may modify associations between VDR genetic variants and cancer risk (38–41), which has not yet been explored for ovarian cancer. Further, risk has not been assessed in relation to GWAS-identified variants linked to lower plasma 25-hydroxyvitamin D [25(OH)D] levels (42–44), a relatively stable indicator of vitamin D status (45).

To explore these gaps, we assessed whether associations between variants presumed to lower VDR bioactivity or contribute to vitamin D insufficiency, and ovarian cancer risk differed by predicted 25(OH)D status in a retrospective case–control study (New England Case–Control study, NECC) and two case–control studies nested within the prospective Nurses’ Health Study (NHS) and NHSII cohorts.

## Materials and Methods

### Study populations

The NHS is a prospective cohort study of 121,700 female registered nurses from 11 US states who were 30–55 years of age in 1976. NHSII began in 1989 and enrolled 116,430 female US registered nurses aged 25–42 from 14 US states. In both cohorts, biennial self-administered questionnaires gathered detailed information on lifestyle, menstrual and reproductive factors, and medical history since baseline. Blood samples were collected from a subset of 32,826 NHS participants in 1989–1990 and from 29,611 NHSII participants from 1996 to 1999. Among women who did not provide a blood sample, buccal cell samples were collected from 29,864 NHS women in 2000–2002 and 29,859 NHSII women in 2004–2006. Eligible cases consisted of women with biospecimen samples diagnosed with pathologically confirmed epithelial ovarian cancer within 4 years before sample collection up to June 1st of either 2009 (buccal cell) or 2011 (blood) in NHSII and 2010 (buccal cell) or 2012 (blood) in NHS with no prior cancer diagnosis except non-melanoma skin cancer. Controls were randomly selected from among women who had at least one ovary and were free of cancer (except non-melanoma skin cancer) up to and including the questionnaire cycle in which the case was diagnosed. Controls were matched to cases by cohort, biospecimen type, age (±1 month), and menopausal status at diagnosis. For participants with blood samples, controls were additionally matched to cases on postmenopausal hormone use (current vs. not current), menopausal status, month/year, time of day, and fasting status at blood collection. Completion of the self-administered questionnaire and submission of the biospecimen was considered to imply informed consent. The NHS and NHSII protocols were approved by the Institutional Review Board of Brigham and Women’s Hospital, Boston, MA, USA.

The NECC Study is a population-based study of ovarian cancer. Women residing in eastern Massachusetts or New Hampshire were recruited during three enrollment phases (Phase 1: 1992–1997, Phase 2: 1998–2002, and Phase 3: 2003–2008), corresponding to three funding periods, the details of which were reported previously (46, 47). Briefly, 3957 women (1,080 from Phase 1, 1,267 from Phase 2, and 1,610 from Phase 3) residing in eastern Massachusetts or New Hampshire with a diagnosis of incident ovarian cancer were identified from hospital tumor boards and statewide cancer registries. Of the 3,083 eligible women, 2,203 agreed to participate. Controls were identified through a combination of random digit dialing, town books, and drivers’ license lists. Exclusion criteria for controls included the inability to be contacted, history of bilateral oophorectomy, language barriers, or relocation outside of the study area. In Phase 1, 420 (72%) of eligible women identified by random digit dialing and 102 (51%) of eligible women identified through town books agreed to participate. In Phase 2 and 3, 4,366 potential controls were identified and 1,578 out of 2,940 eligible women (54%) agreed to participate. Controls were frequency matched to cases by age and study center. At enrollment, participants were asked to recall known and putative ovarian cancer risk factors that occurred ≥1 year prior to diagnosis (for case subjects) or enrollment (control subjects). Over 95% of enrolled participants provided a blood specimen. Each participant provided written informed consent. The Institutional Review Boards of Brigham and Women’s Hospital and Dartmouth Medical School, Hanover, NH, USA approved the study protocol.

### Predicted 25(OH)D scores

We used covariate exposures reported closest to the time of blood collection in NHS and NHSII or at enrollment in NECC to calculate menopause-specific predicted 25(OH)D scores as previously described (20, 48). Briefly, multiple linear regression models to predict 25(OH)D levels within NHS and NHSII were developed and then validated in an independent sample of women with measured 25(OH)D levels (48). Based on these initial models, separate linear regression models were subsequently fit among 2,431 premenopausal and 3,101 postmenopausal women with measured 25(OH)D levels to create menopause-specific predictor scores (20). Predictors of plasma 25(OH)D levels were categorized as follows: race/ethnicity (white, black, Hispanic, Asian, and other), body mass index (BMI; <22.0, 22.0–24.9, 25.0–29.9, 30–34.9, 35+ kg/m^2^), total leisure-time physical activity (<3, 3–8.9, 9–17.9, 18–26.9, 27+ METS/week in NHS/NHSII; 0, quartiles hours/week in NECC), energy-adjusted (49) vitamin D from food (<100, 100–199, 200–299, 300–399, 400+ IU/day), supplemental vitamin D (0, 1–199, 200–399, 400+ IU/day), alcohol intake (0, 0.1–4.9, 5–9.9, 10+ g/day), postmenopausal hormone use (never, past, current, unknown; for postmenopausal women only), and average annual UV-B flux based on state of residence (hereafter simply referred to as UV-B flux; <113, 113, >113 R-B units in NHS/NHSII; all NECC participants reside in states with UV-B flux <113 R-B units). Age (years), season of blood draw (Summer, Fall, Winter, and Spring), and laboratory batch were included in the regression models to account for known variation in 25(OH)D levels. We used the beta values of predictors from the appropriate score to calculate menopause-specific predicted 25(OH)D levels in NHS/NHSII (20). Similarly, we applied these NHS/NHSII-derived beta values as weights for each significant predictor to generate menopause-specific predicted 25(OH)D scores for NECC participants.

### Single nucleotide polymorphism selection and genotyping

We selected commonly studied Single nucleotide polymorphism (SNPs) within the *VDR* gene that either have known or hypothesized functional effects on VDR activity, expression, and/or cancer risk (35). Variants putatively associated with reduced VDR activity were designated as the risk alleles. The rs228570 T allele creates a VDR protein that is three amino acids longer and less transcriptionally active than the protein product of the C allele. The G allele of the *VDR* promoter polymorphism, rs11568820, within the Cdx2 binding site, results in lower binding affinity for the transcription factor. Three SNPs at the 3′ end of the *VDR*: rs1544410, rs7975232, and rs731236 (corresponding RFLPs: *Bsm*I, *Apa*I, and *Taq*I) reside in an area of strong LD, and form several haplotypes of which G-C-A (i.e., baT; 48%) and A-A-C (i.e., BAt; 40%) are most common. These haplotypes are in LD with long and short polyA variable number of tandem repeat (VNTR) alleles in the 3′ untranslated region (UTR) of the gene, respectively, which may alter VDR mRNA stability or translational activity (35). We selected another *VDR* 3′ end SNP rs739837 (RFLP *Bgl*I) that had been associated with advanced breast cancer risk (50), and rs1989969 (−5132 T/C), a promoter SNP that eliminates a potential GATA-1 transcription factor binding site (51). Additionally, we genotyped GWAS-identified SNPs at loci associated with plasma 25(OH)D levels [cytochrome P450, family 2, subfamily R, polypeptide 1 (*CYP2R1*; rs10741657 and rs2060793); NAD synthase 1 (*NADSYN1*)/7-dehydrocholesterol reductase (*DHCR7*; rs1790349 and rs3829251); group-specific component (*GC*; rs2282679, rs4588, and rs7041), which is also commonly known as vitamin D binding protein (VDBP); cytochrome P450, family 24, subfamily A, polypeptide 1 (*CYP24A1*; rs6013897)] (42–44). Three of the GWAS loci also were significantly associated with clinical vitamin D insufficiency (<75 nmol/L) (42). Variants associated with lower plasma 25(OH)D were considered risk alleles.

Existing genotype data on rs11568820, rs1544410, rs2282679, rs228570, and rs7041 were available for NECC Phase 1 and 2 participants from previous analyses (34, 52), whereas genotype data for NHS, NHSII, and NECC Phase 3 participants were newly generated for this project. All 15 *VDR* and vitamin D insufficiency GWAS SNPs were genotyped in NHS and NHSII samples. To increase sample size for haplotype analyses, we additionally genotyped rs1544410, rs4588, rs7041, rs731236, and rs7975232 in NECC Phase 3 samples. Sample sizes for each SNP analyzed are reported in Table S1 in Supplementary Material. Genomic DNA was extracted from buffy coat or buccal cell samples using the QIAamp (Qiagen, Chatsworth, CA, USA) 96-spin blood protocol. DNA from NHS and NHSII participants was whole genome amplified with GE Healthcare Genomiphi (GE Healthcare Bio-Sciences Corp., Piscataway, NJ, USA). Genotyping was performed at the Dana Farber/Harvard Cancer Center High-Throughput Genotyping Core using the 5′ nuclease assay (Taqman, Applied Biosystems, Foster City, CA, USA) on the OpenArray^®^ Real-Time PCR System (Applied Biosystems; NHS/NHSII samples) on amplified DNA or the ABI PRISM^®^ 7900HT Sequence Detection System on genomic DNA (NECC Phase 3 samples). Laboratory personnel were blinded to case–control status, and each plate included replicate samples for quality control, which had 100% concordance.

### 25-Hydroxyvitamin D plasma assay

Existing plasma 25(OH)D levels were available for a subset of NHS and NHSII participants (*N* = 570), as described previously (52, 53). Briefly, case–control sets and samples from the same cohort were assayed together by radioimmunoassay and labeled to mask case–control status. The intra-assay coefficients of variation, based on blinded quality control replicates ranged from 8 to 10%.

### Statistical analysis

We used chi-square tests to assess Hardy–Weinberg equilibrium (HWE) among white controls within NHS/NHSII and NECC. Three of 15 SNPs (rs1989969, rs2060793, and rs739837) were out of HWE among NHS/NHSII participants (*P* < 0.05) and excluded from analyses. We excluded NHS/NHSII samples that failed genotyping of five or more SNPs (15 cases, 41 controls). Among NECC participants, rs1544410 was out of HWE (*P* = 0.004). Review of screen shots for these plates revealed appropriate clustering and so rs1544410 was retained to generate VDR 3′ end haplotypes. The rs1544410 risk allele frequency (RAF) among NECC controls was similar to the RAF among NHS/NHSII controls and the HapMap Phase II + III, Release 28 Caucasian European (CEU) population (Table S1 in Supplementary Material).

We used unconditional logistic regression models adjusted for matching factors and race to calculate odds ratios (ORs) and 95% confidence intervals (CIs). For each SNP, gene dosage effects were modeled by assigning a value of 0, 1, or 2 to a genotype trend variable according to a participant’s number of risk alleles. We estimated frequencies of *VDR* 3′ end and *GC* haplotypes using expectation–maximization-based algorithms and imputed subject-specific expected haplotypes (54, 55) to provide tests of association for individual haplotypes, assuming additive inheritance models. *VDR* 3′ end haplotypes with estimated frequencies <5% were grouped. We derived estimated GC phenotypes based on *GC* haplotype scores and assigned 25(OH)D binding affinity constants based on published estimates [Table 1 in Ref. (56)] to a trend variable. Risk allele counts from the four independent plasma 25(OH)D GWAS loci (rs4588, rs10741567, rs3829251, and rs6013897) were summed to generate a genetic risk score (GRS) of vitamin D insufficiency in NHS/NHSII. Unconditional logistic regression models estimated risk of ovarian cancer associated with the GRS. Linear regression models adjusted for age, cohort, race, and season of blood draw were used to estimate SNP and GRS associations with plasma 25(OH)D levels. Wald tests were used to calculate *P* values for trend.

**Table 1:** **Age and selected age-standardized characteristics of ovarian cancer cases and controls by study population^a^**.

	NHS/NHSII			NECC	
	Case (*N* = 562)	Control (*N* = 1,553)		Case (*N* = 1,821)	Control (*N* = 1,870)
Age (years)^b^	55.0 (7.9)	55.0 (7.9)		52.4 (12.3)	52.3 (12.7)
Ever use of oral contraceptives, %	55	55		53	65
Duration of oral contraceptive use (months)^c^	49.3 (42.8)	54.4 (48.3)		56.0 (56.6)	66.6 (59.6)
Tubal ligation, %	13	21		13	20
Body mass index (kg/m^2^)	25.9 (5.9)	25.2 (4.7)		26.5 (6.3)	26.0 (5.6)
Total activity (hours/week)	3.5 (3.6)	3.4 (3.8)		3.1 (4.9)	3.3 (4.4)
Total vitamin D intake (IU/day)	382 (261)	381 (272)		412 (299)	417 (290)
Alcohol intake (grams/day)	5.9 (9.7)	6.3 (10.8)		6.8 (11.8)	6.7 (10.8)

*^a^Values are means (SD) or percentages and are standardized to the age distribution of the study population*.

*^b^Value is not age-adjusted*.

*^c^Among women who ever used oral contraceptives*.

Predicted 25(OH)D scores were dichotomized at the median values of menopause-specific control distributions in NHS/NHSII and NECC. Women missing predicted 25(OH)D values were excluded from stratified analyses and tests for interaction. Within each study, statistical tests for interaction by predicted 25(OH)D were performed by the Wald test using cross-product terms. DerSimonian and Laird random effects meta-analysis (57) was used to combine results from NHS/NHSII and NECC. Heterogeneity by study and by predicted 25(OH)D strata was calculated using the Q statistic. To calculate a global *P* value for statistical interaction of *VDR* 3′ end haplotypes by predicted 25(OH)D status, we pooled individual-level NHS/NHSII and NECC data, additionally adjusted for study and predicted 25(OH)D scores, and compared models with interaction terms to a model without interaction terms using the likelihood ratio test.

*P* values were based on two-sided tests and considered statistically significant at *P* < 0.05. All analyses were conducted using SAS version 9.3 (SAS Institute Inc., Cary, NC, USA). Power calculations were performed using QUANTO (58).

## Results

A total of 2,383 (562 NHS/NHSII and 1,821 NECC) cases and 3,423 (1,553 NHS/NHSII and 1,870 NECC) controls were available for this analysis. Women ranged in age from 34 to 72 years in NHS/NHSII and from 18 to 79 years in NECC at the time of blood collection. As expected based on the matched designs, cases, and controls were of similar age within each study. On average, NHS/NHSII participants were slightly older than NECC participants at the time of blood collection. Within each study, cases had shorter mean duration of oral contraceptive use and were less likely to have had a tubal ligation than controls. BMI, total leisure-time physical activity, total vitamin D intake (food and supplemental sources), and alcohol consumption were similar between case and control groups. Total vitamin D intake was higher in NECC than in NHS/NHSII (Table 1).

Risk allele frequencies observed in our control groups were comparable to those in the CEU HapMap population (Table S1 in Supplementary Material). Genotyping success rates were ≥95% for all SNPs except rs7975232 in NHS/NHSII (93%) and rs2228570 in NECC (94%). Given high LD between rs1790349 and rs3829251 (*r*^2^ = 0.79) at the *NADSYN1/DHCR7* locus and between rs2282679 and rs4588 (*r*^2^ = 0.95) at the *GC* locus among NHS/NHSII participants, we selected the SNP with the higher genotyping success rate for analysis (rs3829251 and rs4588). We did not observe significant heterogeneity in estimates between NHS/NHSII and NECC in the main effect meta-analysis of SNPs. Of the *VDR* SNPs assessed, we observed a significant 12% increased risk of ovarian cancer associated with each rs7975232 A allele (Table 2). A marginal association between rs2228570 and ovarian cancer risk was also observed (per T allele OR = 1.09, 95% CI = 1.00–1.19; *P*_trend_ = 0.06). The GWAS-identified rs7041 T allele was significantly associated with reduced ovarian cancer risk (per allele OR = 0.92, 95% CI = 0.85–0.99; *P*_trend_ = 0.03). We confirmed that the rs7041 T allele and the vitamin D insufficiency GRS were associated with reduced 25(OH)D levels (*P*_trend_ of 0.005 and <0.0001, respectively) among the subset of NHS/NHSII participants with existing 25(OH)D measurements (*N* = 570). The vitamin D insufficiency GRS was not associated with ovarian cancer risk (data not shown).

**Table 2:** **Association of ovarian cancer with VDR and 25(OH)D GWAS-identified SNPs in the Nurses’ Health Studies, and the New England Case–Control study**.

SNP	Risk allele	Other allele	NHS/NHSIIh		NECC		Meta-analysis		
			Per allele OR (95% CI)^a^	*P*_trend_	Per allele OR (95% CI)^a^	*P*_trend_	Per allele OR (95% CI)^b^	*P*_trend_	*P*_het_*^c^*
**VITAMIN D RECEPTOR POLYMORPHISMS**									
rs11568820	G	A	1.05 (0.88–1.24)	0.61	0.90 (0.78–1.04)	0.14	0.96 (0.83–1.12)	0.60	0.19
rs1544410	A	G	1.09 (0.95–1.25)	0.24	0.98 (0.89–1.08)	0.67	1.02 (0.92–1.12)	0.72	0.23
rs2228570	T	C	1.09 (0.95–1.25)	0.24	1.09 (0.97–1.23)	0.14	1.09 (1.00–1.19)	0.06	0.95
rs731236	C	T	1.12 (0.97–1.29)	0.13	1.02 (0.87–1.20)	0.81	1.07 (0.96–1.20)	0.20	0.42
rs7975232	A	C	1.17 (1.02–1.36)	0.03	1.07 (0.92–1.25)	0.40	1.12 (1.01–1.25)	0.03	0.40
**25(OH)D GWAS LOCI**									
rs4588^d^	T	G	0.99 (0.85–1.15)	0.86	0.95 (0.85–1.05)	0.30	0.96 (0.88–1.05)	0.34	0.65
rs7041	T	G	0.95 (0.83–1.10)	0.51	0.90 (0.82–0.99)	0.03	0.92 (0.85–0.99)	0.03	0.51
rs10741657^d^	G	A	0.96 (0.84–1.11)	0.59	1.14 (1.01–1.30)	0.04	1.05 (0.89–1.24)	0.56	0.08
rs3829251	A	G	0.86 (0.71–1.04)	0.13	–	–	–	–	–
rs6013897	A	T	0.92 (0.77–1.09)	0.32	–	–	–	–	–

*^a^Odds ratios (OR) and 95% confidence intervals (CI) estimated using unconditional logistic regression models adjusted for matching factors and race*.

*^b^DerSimonian–Laird estimators for random effects models were used to combine results from the pooled Nurses’ Health Studies, and the New England Case–Control study datasets*.

*^c^*P*-value for heterogeneity in estimates between studies*.

*^d^rs2282679 was substituted for rs4588 (*r*^2^ = 0.95) and rs2060793 was substituted for rs10741657 (*r*^2^ = 0.88) in a subset of New England Case–Control study participants (950 cases and 1,052 controls) that were genotyped on the iCOGS array (34)*.

We took advantage of the strong LD at the 3′ end of the *VDR* gene (35) to generate haplotype scores that may better capture the effect of an unknown functional variant. Three SNPs at the *VDR* 3′ end (rs1544410–rs7975232–rs731236) formed three common haplotypes and five haplotypes with frequencies <5% that were grouped into a “rare” haplotype variable. Ovarian cancer risk was not associated with the *VDR* 3′ end haplotypes compared to the most common haplotype (G-C-T) in the meta-analysis of NHS/NHSII and NECC (Table 3).

**Table 3:** **Risk of ovarian cancer associated with VDR 3′ end haplotype and GC phenotypes in the Nurses’ Health Studies, and the New England Case–Control study**.

	NHS/NHSII	NECC	Meta-analysis	
	OR (95% CI)^a^	OR (95% CI)^a^	OR (95% CI)^b^	*P*_het_*^c^*
**VDR rs1544410–rs7975232–rs731236 HAPLOTYPES^d^**				
G-C-T	1.00 (ref)	1.00 (ref)	1.00 (ref)	
A-A-C	1.20 (1.01–1.42)	1.02 (0.85–1.22)	1.10 (0.94–1.30)	0.20
G-A-T	1.07 (0.86–1.34)	1.15 (0.89–1.50)	1.10 (0.93–1.31)	0.68
Rare haplotypes	0.89 (0.65–1.22)	1.30 (0.79–2.13)	1.02 (0.72–1.46)	0.21
**GC PHENOTYPES^e^**				
GC2-GC2	1.00 (ref)	1.00 (ref)	1.00 (ref)	
GC2-GC1s	1.07 (0.73–1.58)	1.00 (0.76–1.31)	1.02 (0.82–1.28)	0.77
GC1s-GC1s	1.08 (0.73–1.58)	1.09 (0.83–1.42)	1.08 (0.87–1.35)	0.97
GC2-GC1f	0.98 (0.60–1.60)	0.73 (0.52–1.03)	0.81 (0.61–1.07)	0.33
GC1s-GC1f	1.13 (0.74–1.71)	0.98 (0.73–1.32)	1.03 (0.81–1.31)	0.60
GC1f-GC1f	0.63 (0.28–1.42)	0.89 (0.56–1.41)	0.82 (0.55–1.22)	0.47
*P*_trend_^f^	0.71	0.32	0.31	0.80

*^a^Per haplotype (VDR) or per phenotype (GC) odds ratios (OR) and 95% confidence intervals (CI) estimated using unconditional logistic regression models adjusted for matching factors and race*.

*^b^DerSimonian–Laird estimators for random effects models were used to combine results from the Nurses’ Health Studies, and the New England Case–Control study*.

*^c^*P*-value for heterogeneity in estimates between studies*.

*^d^NHS/NHSII sample size: 485 cases, 1,338 controls; NECC sample size: 575 cases, 610 controls*.

*^e^GC phenotypes derived from haplotype scores of rs4588 and rs7041 (GC2 haplotype: T-T, GC1s haplotype: G-G, GC1f haplotype: G-T); in subset of NECC genotyped on the iCOGS array (34) phenotypes were derived from rs2282679 and rs7041 (GC2 haplotype: C-T, GC1s haplotype: A-G, GC1f haplotype: A-T); NHS/NHSII sample size: 557 cases, 1,523 controls; NECC sample size: 1,587 cases, 1,704 controls*.

*^f^25(OH)D binding affinity constants (*K_a_* × 10^−10^ M) assigned to GC phenotypes for trend test: GC2-GC2, 3.6; GC2-GC1s, 4.8; GC1s-GC1s, 6.0; GC2-GC1f, 7.4; GC1s-GC1f, 8.6; GC1f-GC1f, 11.2 (56)*.

*GC* SNPs rs7041 and rs4588 create amino acid substitutions at positions 416 and 420, respectively, resulting in three major VDBP isoforms (GC2, GC1s, and GC1f) that vary in binding affinity for vitamin D analytes (56). We confirmed that allelic combinations (“GC phenotypes”; see Table 3 footnote) estimated to have higher VDBP binding affinity were positively associated with plasma 25(OH)D levels in NHS/NHSII (*P*_trend_ = 0.003). Estimated GC phenotypes were not associated with ovarian cancer risk in NHS/NHSII, NECC, or the meta-analysis (*P*_trend_ ≥ 0.31; Table 3).

Evidence of effect modification by vitamin D exposure has been observed in prior studies of other cancers. Stronger genetic associations with prostate cancer risk were observed for *VDR* variants among men with vitamin D insufficiency or low sun exposure (38–40). In contrast, the *Bsm*I B allele was somewhat more strongly associated with melanoma risk among participants with higher sun exposure (41). Therefore, we explored the relation between ovarian cancer and individual SNPs, *VDR* 3′ end haplotypes, and estimated GC phenotypes by predicted 25(OH)D strata. Among women with high (above the median) predicted 25(OH)D levels, we observed significant increased risk of ovarian cancer associated with *VDR* variants rs731236 (per allele C OR = 1.31, 95% CI = 1.11–1.55; *P*_trend_ = 0.002) and rs7975232 (per A allele OR = 1.38, 95% CI = 1.17–1.62; *P*_trend_ = 0.0002; Table 4). These SNPs were not associated with ovarian cancer risk among women with low predicted 25(OH)D levels (*P*_heterogeneity_ of 0.009 and 0.006, respectively). Similarly, heterogeneity in ovarian cancer risk by predicted 25(OH)D was observed for the *VDR* 3′ end haplotypes (*P* = 0.009; Table 5). For each A-A-C or G-A-T haplotype possessed by women with high predicted 25(OH)D levels, risk of ovarian cancer significantly increased ~40%. These associations were not observed among women with low predicted 25(OH)D. To determine whether a particular component of the predicted 25(OH)D score accounted for the heterogeneity, we tested effect modification of ovarian cancer associations with rs731236, rs7975232, and *VDR* 3′ end haplotypes by BMI (<25 vs. 25+ kg/m^2^), total vitamin D intake (below vs. above median), total leisure-time physical activity (below vs. above median), menopausal status and HT use (premenopausal, postmenopausal/never HT use, postmenopausal/past HT use, and postmenopausal/current HT use), and alcohol intake (below vs. above median). Except for heterogeneity in the rs731236 association with ovarian cancer risk by alcohol intake (*P* = 0.03), in which the increased risk was restricted to women with higher than the median alcohol intake, we did not observe significant effect modification by predicted 25(OH)D score components (data not shown). The *GC* rs7041 T allele was associated with reduced ovarian cancer risk among women with low predicted 25(OH)D (*P*_trend_ = 0.03), but the association was not significantly different from that of women with high predicted 25(OH)D (*P*_heterogeneity_ = 0.39; Table 4). Estimated GC phenotypes were not associated with ovarian cancer risk regardless of predicted 25(OH)D status (Table 5).

**Table 4:** **Association of ovarian cancer with VDR and 25(OH)D GWAS-identified SNPs by predicted 25(OH)D status^a^**.

SNP	Risk allele	Other allele	Below median predicted 25(OH)D			Above median predicted 25(OH)D			*P*_het_ *^c^*
			Cases/controls	Per allele OR (95% CI)^b^	*P*_trend_	Cases/controls	Per allele OR (95% CI)^b^	*P*_trend_	
**VITAMIN D RECEPTOR POLYMORPHISMS**									
rs11568820	G	A	883/1,285	0.94 (0.79–1.13)	0.52	617/1,019	0.90 (0.75–1.09)	0.28	0.72
rs1544410	A	G	1,166/1,537	0.97 (0.86–1.08)	0.54	912/1,377	1.12 (0.94–1.33)	0.21	0.16
rs2228570	T	C	877/1,286	1.09 (0.91–1.29)	0.35	614/1,009	1.15 (0.99–1.33)	0.07	0.78
rs731236	C	T	549/935	0.94 (0.78–1.14)	0.56	472/894	1.31 (1.11–1.55)	0.002	0.009
rs7975232	A	C	520/885	0.98 (0.83–1.16)	0.82	481/848	1.38 (1.17–1.62)	0.0002	0.006
**25(OH)D GWAS LOCI**									
rs4588^d^	T	G	1,088/1,504	0.97 (0.85–1.09)	0.58	863/1,342	0.93 (0.81–1.06)	0.29	0.74
rs7041	T	G	1,087/1,500	0.88 (0.79–0.99)	0.03	857/1,344	0.94 (0.83–1.07)	0.35	0.39
rs10741657^d^	G	A	791/1,226	1.06 (0.77–1.47)^e^	0.71	552/1,965	0.99 (0.85–1.16)	0.90	0.72^e^
rs3829251^f^	A	G	269/692	0.81 (0.60–1.07)	0.14	198/549	0.98 (0.70–1.36)	0.88	0.35
rs6013897^f^	A	T	268/694	1.00 (0.78–1.30)	0.98	198/546	0.89 (0.66–1.21)	0.46	0.61

*^a^DerSimonian–Laird estimators for random effects models were used to combine results from the pooled Nurses’ Health Studies, and the New England Case–Control study datasets*.

*^b^Odds ratios (OR) and 95% confidence intervals (CI) estimated using unconditional logistic regression models adjusted for matching factors and race*.

*^c^*P*-value for heterogeneity by predicted 25(OH)D levels*.

*^d^rs2282679 was substituted for rs4588 (*r*^2^ = 0.95) and rs2060793 was substituted for rs10741657 (*r*^2^ = 0.88) in a subset of the New England Case–Control study participants (950 cases and 1,052 controls) genotyped on the iCOGS array (34)*.

*^e^*P* < 0.05 for test for heterogeneity between the Nurses’ Health Studies, and the New England Case–Control study estimates*.

*^f^Estimates only available from the Nurses’ Health Studies*.

**Table 5:** **Risk of ovarian cancer associated with. VDR 3′ end haplotype and GC phenotypes by predicted 25(OH)D status^a^**.

	Below median predicted 25(OH)D		Above median predicted 25(OH)D		*P*_het_ *^c^*
	OR (95% CI)^b^	*P*_trend_	OR (95% CI)^b^	*P*_trend_	
**VDR rs1544410–rs7975232–rs731236 HAPLOTYPES^d^**					
G-C-T	1.00 (ref)		1.00 (ref)		0.009
A-A-C	0.92 (0.73–1.14)	0.44	1.41 (1.16–1.71)	0.0005	
G-A-T	1.02 (0.79–1.32)	0.89	1.37 (1.05–1.78)	0.02	
Rare haplotypes	0.87 (0.52–1.45)	0.59	1.34 (0.88–2.04)	0.18	
**GC PHENOTYPES^e^**					
GC2–GC2	1.00 (ref)		1.00 (ref)		
GC2–GC1s	1.38 (0.95–2.01)		0.87 (0.35–2.19)^f^		
GC1s–GC1s	1.32 (0.96–1.82)		0.89 (0.63–1.26)		
GC2–GC1f	0.99 (0.67–1.47)		0.61 (0.39–0.96)		
GC1s–GC1f	1.15 (0.81–1.63)		1.09 (0.56–2.12)		
GC1f–GC1f	0.82 (0.38–1.76)		0.77 (0.40–1.47)		
Ptrend^g^	0.10		0.52		0.12

*^a^DerSimonian–Laird estimators for random effects models were used to combine results from the Nurses’ Health Studies, and the New England Case–Control study*.

*^b^Per haplotype (VDR) or per phenotype (GC) odds ratios (OR) and 95% confidence intervals (CI) estimated using unconditional logistic regression models adjusted for matching factors and race*.

*^c^*P*-value for heterogeneity by predicted 25(OH)D levels*.

*^d^Below median predicted 25(OH)D sample size: 496 cases, 842 controls; above median predicted 25(OH)D sample size: 435 cases, 808 controls*.

*^e^GC phenotypes derived from haplotype scores of rs4588 and rs7041 (GC2 haplotype: T-T, GC1s haplotype: G-G, and GC1f haplotype: G-T); in subset of NECC genotyped on the iCOGS array (34) phenotypes were derived from rs2282679 and rs7041 (GC2 haplotype: C-T, GC1s haplotype: A-G, and GC1f haplotype: A-T); below median predicted 25(OH)D sample size: 1,084 cases, 1,487 controls; above median predicted 25(OH)D sample size: 854 cases, 1,334 controls*.

*^f^*P* < 0.05 for test for heterogeneity between the Nurses’ Health Studies, and the New England Case–Control study estimates*.

*^g^25(OH)D binding affinity constants (K_a_ × 10^−10^ M) assigned to GC phenotypes for trend test: GC2–GC2, 3.6; GC2–GC1s, 4.8; GC1s–GC1s, 6.0; GC2–GC1f, 7.4; GC1s–GC1f, 8.6; GC1f–GC1f, 11.2 (56)*.

## Discussion

In this study, we examined whether the vitamin D status of individuals modifies genetic associations between *VDR* variants and ovarian cancer risk. Our results provide some evidence that genetic variation at the 3′ end of the *VDR* gene (rs731236, rs7975232, 3′ end haplotypes) may influence ovarian cancer risk among women with higher predicted 25(OH)D levels, but not among women with lower levels. This also is the first report to assess whether vitamin D insufficiency GWAS loci are associated with risk. Based on the anti-proliferative and pro-apoptotic effects of vitamin D on ovarian cancer cell lines (1–4), the rs7041 vitamin D insufficiency risk (T) allele was unexpectedly associated with reduced risk of ovarian cancer in our datasets. Outside of the *VDR* 3′end genetic variants, we did not observe effect modification by predicted 25(OH)D status for the other examined variants.

Overall, we observed that some, but not all, genetic variation at the 3′ end of the *VDR* gene is associated with a modestly increased risk of ovarian cancer. Known vitamin D pathway genes do not appear to reside at the nine susceptibility loci newly identified by ovarian cancer GWAS studies conducted among women from North America and the UK (31–34). In a US GWAS of ovarian cancer (*n* = 1,814 cases and 1,867 controls from four studies), there was no association between the 3′ end haplotypes and ovarian cancer risk (unpublished data). However, in our study, we only observed a positive association for individuals with higher predicted 25(OH)D status. Since a substantial portion of the US and UK populations have insufficient vitamin D levels (59, 60), it may be difficult to observe genetic associations without considering the vitamin D status of women. The biologic implications of our results are not entirely clear as we observed heterogeneity by predicted 25(OH)D status for variants with unknown functional significance. *VDR* 3′ end haplotypes are in strong LD with regulatory elements in the 3′ UTR, which may alter VDR mRNA stability or translational activity. However, reported directions of association between 3′ end haplotypes and VDR expression and/or activity have been inconsistent, which may be due to tissue-specific regulation (35). Alternatively, the *VDR* 3′ variants could be in LD with variants influencing activity and/or expression of neighboring genes such as histone deacetylase 7 (*HDAC7*), which begins 21.6 kb downstream of the *VDR* gene. HDAC7 has been shown to attenuate 1,25(OH)D-mediated gene transcription in malignant breast cells (61). Investigating the influence of *VDR* 3′ end variants on expression and/or activity of the *VDR* and neighboring genes in ovarian cells as well as potential vitamin D activation of the receptor may provide insight on the relation with ovarian cancer risk.

An experimental study demonstrated that 1,25(OH)D-stimulated VDR activity differed by naturally occurring rs2228570 genotype in peripheral blood mononuclear cells *in vitro* (62). The T allele exhibited a dose-dependent effect on the half-maximal activity of 1,25(OH)D, in which higher concentrations were required to obtain similar inhibition of cell proliferation. However, the polymorphism was not associated with maximal 1,25(OH)D-mediated growth inhibition. 1,25(OH)D half-maximal and maximal growth inhibition did not differ by rs1544410, rs731236, rs7975232 genotypes, or *VDR* 3′ end haplotypes (62). The authors noted that the lack of heterogeneity by *VDR* 3′ end haplotypes potentially could have been due to small sample size (62), which may be true for the individual variants as well. Likewise, as common variants often exhibit weak effects on associated phenotypes, our analysis was not sufficiently powered to detect small differences in ovarian cancer risk by genotype, which may have contributed to inconsistency between our study and the experimental study. For example, based on our observed estimates within predicted 25(OH)D strata, our power to detect significant relative ORs of ~1.06 for rs2228570 and ~1.15 for rs1544410 were 10 and 43%, respectively. Further, while the predicted 25(OH)D score is significantly positively correlated with plasma 25(OH)D levels (48), we cannot estimate absolute levels to determine which subgroup of women are exposed to half-maximal concentrations of 25(OH)D or 1,25(OH)D in order to observe potential differences by genotype. Lastly, growth inhibition exhibited by 1,25(OH)D on peripheral blood mononuclear cells may differ from 1,25(OH)D-mediated inhibition of normal and/or malignant ovarian cells.

We hypothesized that validated GWAS variants associated with lower plasma 25(OH)D levels would result in a lower lifetime average plasma 25(OH)D levels, increasing risk of ovarian cancer. Individual vitamin D insufficiency variants and the GRS were associated with plasma 25(OH)D in the expected direction among the subgroup of NHS/NHSII women with measured levels, but were not associated with ovarian cancer risk. Contrary to expectations, the rs7041 T allele was associated with reduced ovarian cancer risk (*P* = 0.03). The association could be due to chance considering the number of statistical tests performed in this study. Another possibility is that because rs7041, in combination with rs4588, forms three major VDBP isoforms (GC2, GC1s, and GC1f), and the rs7041 T allele codes for isoforms with both the lowest (GC2) and highest (GC1f) binding affinity (56), our study population may have a higher prevalence of the GC1f compared to the GC2 isoform. However, consistent with prior reports of race-specific frequencies (63), the frequency of GC2 (~0.28) was higher than that of GC1f (~0.15) among our predominantly white study populations. The trend variable of estimated GC phenotypes ordered by increasing binding affinity was associated with higher plasma 25(OH)D, but not ovarian cancer risk in our study. Stratifying by predicted 25(OH)D did not identify ovarian cancer associations with individual GWAS variants nor with estimated GC phenotypes. While we adjusted our analyses for self-reported race, given racial differences in VDBP isoform frequencies (63), and in risk of ovarian cancer (64), we cannot exclude the possibility that underlying population stratification may have confounded our results. Estimates for all of our analyses were similar when we excluded women of self-reported non-European ancestry (NHS/NHSII: 29 cases, 74 controls; NECC: 89 cases, 57 controls).

Our study has several strengths and limitations. While our study benefited from a relatively large sample size for the main effects on at least a subset of examined SNPs, power to detect heterogeneity by predicted 25(OH)D status was generally limited. Moreover, the numerous hypotheses tested relating to SNP main effects, haplotypes, predicted 25(OH)D strata, and interactions increased the likelihood of observing false positive results. Even so, increased ovarian cancer risk associated with rs7975232 and the A-A-C 3′ end haplotype at the *VDR* locus among women with higher predicted 25(OH)D scores remained significant after adjusting the significance level using a Bonferroni correction (0.05/54 = 0.0009). The risk associated with *VDR* variant rs731236 among this same subgroup of women also remained significant using the less conservative False Discovery Rate procedure (65). Our analyses were aided by the predominantly white study populations, but lacked ancestry informative markers to control for potential population stratification. Further, our results may not be generalizable to non-white populations, particularly for VDBP isoforms, the frequencies of which are known to vary substantially by race (63). Use of the questionnaire-based predicted 25(OH)D score made our large study feasible. In general, the heterogeneity observed by predicted 25(OH)D status did not appear to be driven by a single component of the score predicting 25(OH)D, but rather by the score in its entirety. However, we are unable to directly translate the predicted 25(OH)D score into absolute 25(OH)D levels. Future follow-up studies with measured plasma 25(OH)D levels on a larger study population could not only be used to validate our results, but also determine the most biologically relevant vitamin D forms [e.g., 25(OH)D or 1,25(OH)D] to detect differences in genetic associations with ovarian cancer risk. Additionally, although the predicted 25(OH)D score has been validated in NHS/NHSII, we were unable to assess the performance of the score among NECC participants due to the absence of measured 25(OH)D levels. While the geographic distribution of NHS/NHSII participants differs from that of NECC, many NHS/NHSII women reside in the Northeast and are of a similar age distribution, which may improve the generalizability of the score. Finally, our sample size was not sufficient to assess genetic associations by histologic subtypes.

In summary, we observed heterogeneity in genetic associations with ovarian cancer risk by predicted 25(OH)D status that was limited to variation at the 3′ end of the *VDR* gene. Our results indicate that genetic associations may be missed if 25(OH)D status is not considered, potentially contributing to inconsistency in the literature. Larger studies assessing heterogeneity in ovarian cancer risk by vitamin D status associated with *VDR* variants are required to validate our results. If resources are available, use of plasma 25(OH)D levels instead of predicted 25(OH)D scores may provide additional information on whether genetic variants are likely to influence risk over a particular threshold or range of 25(OH)D. Moreover, functional studies assessing the influence of the VDR 3′ end variants on expression and activity of VDR and neighboring genes in ovarian tissue may shed light on the degree of involvement of the vitamin D pathway compared to alternate pathways that may involve other genes in the region.

## Conflict of Interest Statement

The authors declare that the research was conducted in the absence of any commercial or financial relationships that could be construed as a potential conflict of interest.

## Supplementary Material

The Supplementary Material for this article can be found online at http://www.frontiersin.org/Journal/10.3389/fonc.2014.00286/abstract

## References

[1] Saunders DE Christensen C Wappler NL Schultz JF Lawrence WD Malviya VK Inhibition of c-myc in breast and ovarian carcinoma cells by 1,25-dihydroxyvitamin D3, retinoic acid and dexamethasone. Anticancer Drugs (1993) 4(2):201–8. 10.1097/00001813-199304000-00012 8490200

[2] Ahonen MH Zhuang YH Aine R Ylikomi T Tuohimaa P. Androgen receptor and vitamin D receptor in human ovarian cancer: growth stimulation and inhibition by ligands. Int J Cancer (2000) 86(1):40–6. 10.1002/(SICI)1097-0215(20000401)86:1<40::AID-IJC6>3.0.CO;2-E 10728592

[3] Li P Li C Zhao X Zhang X Nicosia SV Bai W. p27(Kip1) stabilization and G(1) arrest by 1,25-dihydroxyvitamin D(3) in ovarian cancer cells mediated through down-regulation of cyclin E/cyclin-dependent kinase 2 and Skp1-Cullin-F-box protein/Skp2 ubiquitin ligase. J Biol Chem (2004) 279(24):25260–7. 10.1074/jbc.M311052200 15075339

[4] Jiang F Bao J Li P Nicosia SV Bai W. Induction of ovarian cancer cell apoptosis by 1,25-dihydroxyvitamin D3 through the down-regulation of telomerase. J Biol Chem (2004) 279(51):53213–21. 10.1074/jbc.M410395200 15485861

[5] Lange TS Stuckey AR Robison K Kim KK Singh RK Raker CA Effect of a vitamin D(3) derivative (B3CD) with postulated anti-cancer activity in an ovarian cancer animal model. Invest New Drugs (2010) 28(5):543–53. 10.1007/s10637-009-9284-y 19582372 PMC2904825

[6] Kawar N Maclaughlan S Horan TC Uzun A Lange TS Kim KK PT19c, another nonhypercalcemic vitamin D2 derivative, demonstrates antitumor efficacy in epithelial ovarian and endometrial cancer models. Genes Cancer (2013) 4(11–12):524–34. 10.1177/1947601913507575 24386512 PMC3877664

[7] Saunders DE Christensen C Lawrence WD Malviya VK Malone JM Williams JR Receptors for 1,25-dihydroxyvitamin D3 in gynecologic neoplasms. Gynecol Oncol (1992) 44(2):131–6. 10.1016/0090-8258(92)90028-H 1312051

[8] Villena-Heinsen C Meyberg R Axt-Fliedner R Reitnauer K Reichrath J Friedrich M. Immunohistochemical analysis of 1,25-dihydroxyvitamin-D3-receptors, estrogen and progesterone receptors and Ki-67 in ovarian carcinoma. Anticancer Res (2002) 22(4):2261–7. 12174912

[9] Thill M Fischer D Kelling K Hoellen F Dittmer C Hornemann A Expression of vitamin D receptor (VDR), cyclooxygenase-2 (COX-2) and 15-hydroxyprostaglandin dehydrogenase (15-PGDH) in benign and malignant ovarian tissue and 25-hydroxycholecalciferol (25(OH2)D3) and prostaglandin E2 (PGE2) serum level in ovarian cancer patients. J Steroid Biochem Mol Biol (2010) 121(1–2):387–90. 10.1016/j.jsbmb.2010.03.049 20304053

[10] Friedrich M Rafi L Mitschele T Tilgen W Schmidt W Reichrath J. Analysis of the vitamin D system in cervical carcinomas, breast cancer and ovarian cancer. Recent Results Cancer Res (2003) 164:239–46. 10.1007/978-3-642-55580-0_17 12899526

[11] Anderson MG Nakane M Ruan X Kroeger PE Wu-Wong JR. Expression of VDR and CYP24A1 mRNA in human tumors. Cancer Chemother Pharmacol (2006) 57(2):234–40. 10.1007/s00280-005-0059-7 16180015

[12] Lefkowitz ES Garland CF. Sunlight, vitamin D, and ovarian cancer mortality rates in US women. Int J Epidemiol (1994) 23(6):1133–6. 10.1093/ije/23.6.1133 7721513

[13] Garland CF Mohr SB Gorham ED Grant WB Garland FC. Role of ultraviolet B irradiance and vitamin D in prevention of ovarian cancer. Am J Prev Med (2006) 31(6):512–4. 10.1016/j.amepre.2006.08.018 17169713

[14] Grant WB. The likely role of vitamin D from solar ultraviolet-B irradiance in increasing cancer survival. Anticancer Res (2006) 26(4A):2605–14. 16886670

[15] Grant WB. A meta-analysis of second cancers after a diagnosis of nonmelanoma skin cancer: additional evidence that solar ultraviolet-B irradiance reduces the risk of internal cancers. J Steroid Biochem Mol Biol (2007) 103(3–5):668–74. 10.1016/j.jsbmb.2006.12.030 17208438

[16] Grant WB. An ecological study of cancer mortality rates in the United States with respect to solar ultraviolet-B doses, smoking, alcohol consumption and urban/rural residence. Dermatoendocrinol (2010) 2(2):68–76. 10.4161/derm.2.2.13812 21547102 PMC3081680

[17] Tran B Jordan SJ Lucas R Webb PM Neale R. Association between ambient ultraviolet radiation and risk of epithelial ovarian cancer. Cancer Prev Res (Phila) (2012) 5(11):1330–6. 10.1158/1940-6207.CAPR-12-0279 23034146

[18] Bodelon C Cushing-Haugen KL Wicklund KG Doherty JA Rossing MA. Sun exposure and risk of epithelial ovarian cancer. Cancer Causes Control (2012) 23(12):1985–94. 10.1007/s10552-012-0076-x 23065074 PMC3499637

[19] Lin SW Wheeler DC Park Y Cahoon EK Hollenbeck AR Freedman DM Prospective study of ultraviolet radiation exposure and risk of cancer in the United States. Int J Cancer (2012) 131(6):E1015–23. 10.1002/ijc.27619 22539073 PMC3402606

[20] Prescott J Bertrand KA Poole EM Rosner BA Tworoger SS. Surrogates of long-term vitamin d exposure and ovarian cancer risk in two prospective cohort studies. Cancers (Basel) (2013) 5(4):1577–600. 10.3390/cancers5041577 24351671 PMC3875955

[21] Merritt MA Cramer DW Vitonis AF Titus LJ Terry KL. Dairy foods and nutrients in relation to risk of ovarian cancer and major histological subtypes. Int J Cancer (2013) 132(5):1114–24. 10.1002/ijc.27701 22740148 PMC3495088

[22] Goodman MT Wu AH Tung KH McDuffie K Kolonel LN Nomura AM Association of dairy products, lactose, and calcium with the risk of ovarian cancer. Am J Epidemiol (2002) 156(2):148–57. 10.1093/aje/kwf022 12117706

[23] Kushi LH Mink PJ Folsom AR Anderson KE Zheng W Lazovich D Prospective study of diet and ovarian cancer. Am J Epidemiol (1999) 149(1):21–31. 10.1093/oxfordjournals.aje.a009723 9883790

[24] Koralek DO Bertone-Johnson ER Leitzmann MF Sturgeon SR Lacey JV Jr Schairer C Relationship between calcium, lactose, vitamin D, and dairy products and ovarian cancer. Nutr Cancer (2006) 56(1):22–30. 10.1207/s15327914nc5601_4 17176214

[25] Bidoli E La Vecchia C Talamini R Negri E Parpinel M Conti E Micronutrients and ovarian cancer: a case-control study in Italy. Ann Oncol (2001) 12(11):1589–93. 10.1023/A:1013124112542 11822759

[26] Salazar-Martinez E Lazcano-Ponce EC Gonzalez Lira-Lira G Escudero-De los Rios P Hernandez-Avila M. Nutritional determinants of epithelial ovarian cancer risk: a case-control study in Mexico. Oncology (2002) 63(2):151–7. 10.1159/000063814 12239450

[27] Genkinger JM Hunter DJ Spiegelman D Anderson KE Arslan A Beeson WL Dairy products and ovarian cancer: a pooled analysis of 12 cohort studies. Cancer Epidemiol Biomarkers Prev (2006) 15(2):364–72. 10.1158/1055-9965.EPI-05-0484 16492930

[28] Zheng W Danforth KN Tworoger SS Goodman MT Arslan AA Patel AV Circulating 25-hydroxyvitamin D and risk of epithelial ovarian cancer: cohort consortium vitamin D pooling project of rarer cancers. Am J Epidemiol (2010) 172(1):70–80. 10.1093/aje/kwq118 20562186 PMC2892541

[29] Toriola AT Surcel HM Agborsangaya C Grankvist K Tuohimaa P Toniolo P Serum 25-hydroxyvitamin D and the risk of ovarian cancer. Eur J Cancer (2010) 46(2):364–9. 10.1016/j.ejca.2010.05.019 19713101

[30] Toriola AT Surcel HM Calypse A Grankvist K Luostarinen T Lukanova A Independent and joint effects of serum 25-hydroxyvitamin D and calcium on ovarian cancer risk: a prospective nested case-control study. Eur J Cancer (2010) 46(15):2799–805. 10.1016/j.ejca.2010.05.019 20601305

[31] Song H Ramus SJ Tyrer J Bolton KL Gentry-Maharaj A Wozniak E A genome-wide association study identifies a new ovarian cancer susceptibility locus on 9p22.2. Nat Genet (2009) 41(9):996–1000. 10.1038/ng.424 19648919 PMC2844110

[32] Goode EL Chenevix-Trench G Song H Ramus SJ Notaridou M Lawrenson K A genome-wide association study identifies susceptibility loci for ovarian cancer at 2q31 and 8q24. Nat Genet (2010) 42(10):874–9. 10.1038/ng.668 20852632 PMC3020231

[33] Bolton KL Tyrer J Song H Ramus SJ Notaridou M Jones C Common variants at 19p13 are associated with susceptibility to ovarian cancer. Nat Genet (2010) 42(10):880–4. 10.1038/ng.666 20852633 PMC3125495

[34] Pharoah PD Tsai YY Ramus SJ Phelan CM Goode EL Lawrenson K GWAS meta-analysis and replication identifies three new susceptibility loci for ovarian cancer. Nat Genet (2013) 45(4):e1–2. 10.1038/ng.2564 23535730 PMC3693183

[35] Uitterlinden AG Fang Y Van Meurs JB Pols HA Van Leeuwen JP. Genetics and biology of vitamin D receptor polymorphisms. Gene (2004) 338(2):143–56. 10.1016/j.gene.2004.05.014 15315818

[36] Xu H Li S Qiu JQ Gao XL Zhang P Yang YX. The VDR gene FokI polymorphism and ovarian cancer risk. Tumour Biol (2013) 34(6):3309–16. 10.1007/s13277-013-0826-8 24078452

[37] Liu Y Li C Chen P Li X Li M Guo H Polymorphisms in the vitamin D receptor (VDR) and the risk of ovarian cancer: a meta-analysis. PLoS One (2013) 8(6):e66716. 10.1371/journal.pone.0066716 23826116 PMC3691226

[38] Mikhak B Hunter DJ Spiegelman D Platz EA Hollis BW Giovannucci E. Vitamin D receptor (VDR) gene polymorphisms and haplotypes, interactions with plasma 25-hydroxyvitamin D and 1,25-dihydroxyvitamin D, and prostate cancer risk. Prostate (2007) 67(9):911–23. 10.1002/pros.20570 17440943

[39] Rukin NJ Luscombe C Moon S Bodiwala D Liu S Saxby MF Prostate cancer susceptibility is mediated by interactions between exposure to ultraviolet radiation and polymorphisms in the 5’ haplotype block of the vitamin D receptor gene. Cancer Lett (2007) 247(2):328–35. 10.1016/j.canlet.2006.05.012 16815628

[40] Ahn J Albanes D Berndt SI Peters U Chatterjee N Freedman ND Vitamin D-related genes, serum vitamin D concentrations and prostate cancer risk. Carcinogenesis (2009) 30(5):769–76. 10.1093/carcin/bgp055 19255064 PMC2675652

[41] Mandelcorn-Monson R Marrett L Kricker A Armstrong BK Orlow I Goumas C Sun exposure, vitamin D receptor polymorphisms FokI and BsmI and risk of multiple primary melanoma. Cancer Epidemiol (2011) 35(6):e105–10. 10.1016/j.canep.2011.03.003 21612999 PMC3182291

[42] Wang TJ Zhang F Richards JB Kestenbaum B van Meurs JB Berry D Common genetic determinants of vitamin D insufficiency: a genome-wide association study. Lancet (2010) 376(9736):180–8. 10.1016/S0140-6736(10)60588-0 20541252 PMC3086761

[43] Ahn J Yu K Stolzenberg-Solomon R Simon KC McCullough ML Gallicchio L Genome-wide association study of circulating vitamin D levels. Hum Mol Genet (2010) 19(13):2739–45. 10.1093/hmg/ddq155 20418485 PMC2883344

[44] Lasky-Su J Lange N Brehm JM Damask A Soto-Quiros M Avila L Genome-wide association analysis of circulating vitamin D levels in children with asthma. Hum Genet (2012) 131(9):1495–505. 10.1007/s00439-012-1185-z 22673963 PMC3648789

[45] Adams JS Clemens TL Parrish JA Holick MF. Vitamin-D synthesis and metabolism after ultraviolet irradiation of normal and vitamin-D-deficient subjects. N Engl J Med (1982) 306(12):722–5. 10.1056/NEJM198203253061206 7038486

[46] Terry KL De Vivo I Titus-Ernstoff L Sluss PM Cramer DW. Genetic variation in the progesterone receptor gene and ovarian cancer risk. Am J Epidemiol (2005) 161(5):442–51. 10.1093/aje/kwi064 15718480 PMC1380205

[47] Harris HR Cramer DW Vitonis AF DePari M Terry KL. Folate, vitamin B(6), vitamin B(12), methionine and alcohol intake in relation to ovarian cancer risk. Int J Cancer (2012) 131(4):E518–29. 10.1002/ijc.26455 21953625 PMC3288483

[48] Bertrand KA Giovannucci E Liu Y Malspeis S Eliassen AH Wu K Determinants of plasma 25-hydroxyvitamin D and development of prediction models in three US cohorts. Br J Nutr (2012) 108(10):1889–96. 10.1017/S0007114511007409 22264926 PMC3346859

[49] Willett WC Howe GR Kushi LH. Adjustment for total energy intake in epidemiologic studies. Am J Clin Nutr (1997) 65(4 Suppl):1220S–8S. 9094926 10.1093/ajcn/65.4.1220S

[50] John EM Schwartz GG Koo J Wang W Ingles SA. Sun exposure, vitamin D receptor gene polymorphisms, and breast cancer risk in a multiethnic population. Am J Epidemiol (2007) 166(12):1409–19. 10.1093/aje/kwm259 17934201

[51] Kidd LC Paltoo DN Wang S Chen W Akereyeni F Isaacs W Sequence variation within the 5’ regulatory regions of the vitamin D binding protein and receptor genes and prostate cancer risk. Prostate (2005) 64(3):272–82. 10.1002/pros.20204 15717311

[52] Tworoger SS Gates MA Lee IM Buring JE Titus-Ernstoff L Cramer D Polymorphisms in the vitamin D receptor and risk of ovarian cancer in four studies. Cancer Res (2009) 69(5):1885–91. 10.1158/0008-5472.CAN-08-3515 19223536 PMC2666963

[53] Hollis BW. Quantitation of 25-hydroxyvitamin D and 1,25-dihydroxyvitamin D by radioimmunoassay using radioiodinated tracers. Methods Enzymol (1997) 282:174–86. 10.1016/S0076-6879(97)82106-4 9330287

[54] Zaykin DV Westfall PH Young SS Karnoub MA Wagner MJ Ehm MG. Testing association of statistically inferred haplotypes with discrete and continuous traits in samples of unrelated individuals. Hum Hered (2002) 53(2):79–91. 10.1159/000057986 12037407

[55] Kraft P Cox DG Paynter RA Hunter D De Vivo I. Accounting for haplotype uncertainty in matched association studies: a comparison of simple and flexible techniques. Genet Epidemiol (2005) 28(3):261–72. 10.1002/gepi.20061 15637718

[56] Arnaud J Constans J. Affinity differences for vitamin D metabolites associated with the genetic isoforms of the human serum carrier protein (DBP). Hum Genet (1993) 92(2):183–8. 10.1007/BF00219689 8370586

[57] DerSimonian R Laird N. Meta-analysis in clinical trials. Control Clin Trials (1986) 7(3):177–88. 10.1016/0197-2456(86)90046-2 3802833

[58] Gauderman W Morrison J. QUANTO 1.1: A Computer Program for Power and Sample Size Calculations for Genetic-Epidemiology Studies. (2006). Available from: http://hydra.usc.edu/gxe

[59] Ginde AA Liu MC Camargo CA Jr. Demographic differences and trends of vitamin D insufficiency in the US population, 1988-2004. Arch Intern Med (2009) 169(6):626–32. 10.1001/archinternmed.2008.604 19307527 PMC3447083

[60] Prentice A. Vitamin D deficiency: a global perspective. Nutr Rev (2008) 66(10 Suppl 2):S153–64. 10.1111/j.1753-4887.2008.00100.x 18844843

[61] Malinen M Saramaki A Ropponen A Degenhardt T Vaisanen S Carlberg C. Distinct HDACs regulate the transcriptional response of human cyclin-dependent kinase inhibitor genes to Trichostatin A and 1alpha, 25-dihydroxyvitamin D3. Nucleic Acids Res (2008) 36(1):121–32. 10.1093/nar/gkm913 17999998 PMC2248733

[62] Colin EM Weel AE Uitterlinden AG Buurman CJ Birkenhager JC Pols HA Consequences of vitamin D receptor gene polymorphisms for growth inhibition of cultured human peripheral blood mononuclear cells by 1, 25-dihydroxyvitamin D3. Clin Endocrinol (Oxf) (2000) 52(2):211–6. 10.1046/j.1365-2265.2000.00909.x 10671949

[63] Kamboh MI Ferrell RE. Ethnic variation in vitamin D-binding protein (GC): a review of isoelectric focusing studies in human populations. Hum Genet (1986) 72(4):281–93. 10.1007/BF00290950 3516862

[64] Tortolero-Luna G Mitchell MF. The epidemiology of ovarian cancer. J Cell Biochem Suppl (1995) 23:200–7. 10.1002/jcb.240590927 8747397

[65] Benjamini Y Drai D Elmer G Kafkafi N Golani I. Controlling the false discovery rate in behavior genetics research. Behav Brain Res (2001) 125(1–2):279–84. 10.1016/S0166-4328(01)00297-2 11682119

