# Supplementary material for: Evidence of Differential Effects of Vitamin D Receptor Variants on Epithelial Ovarian Cancer Risk by Predicted Vitamin D Status
Source: Front Oncol. 2014 Oct 20;4:286. doi: 10.3389/fonc.2014.00286 (PMC4202710; doi:10.3389/fonc.2014.00286)
Supplement: Supplementary file 1 [file Table1.DOCX]

**Supplementary Table 1 | SNP information**

|  |  |  |  |  |  | Total Case / Control N | | Risk allele frequencies | | |
| --- | --- | --- | --- | --- | --- | --- | --- | --- | --- | --- |
| SNP | Chr | Position^a^ | RFLP  designation  or nearest gene | Risk allele  (restriction  site allele) | Other allele  (restriction  site allele) | NHS/  NHSII | NECC | NHS/  NHSII^b^ | NECC^b^ | PhaseII+III  CEU |
| *Vitamin D Receptor* | | | | | | | | | | |
| rs11568820 | 12 | 46,588,812 | Cdx2 | G | A | 554/1,531 | 1,120/1,157 | 0.80 | 0.81 | 0.80 |
| rs1544410 | 12 | 46,526,102 | BsmI | A (B) | G (b) | 559/1,534 | 1,739/1,794 | 0.40 | 0.40 | 0.44 |
| rs2228570 | 12 | 46,559,162 | FokI | T (f) | C (F) | 558/1,543 | 1,104/1,135 | 0.39 | 0.38 | 0.41^c^ |
| rs731236 | 12 | 46,525,024 | TaqI | C (t) | T (T) | 551/1,519 | 611/643 | 0.38 | 0.38 | 0.44 |
| rs7975232 | 12 | 46,525,104 | ApaI | A (A) | C (a) | 498/1,386 | 636/659 | 0.53 | 0.51 | 0.57 |
| *25(OH)D GWAS loci* | | | | | | | | | | |
| rs4588^d^ | 4 | 72,837,187 | GC | T | G | 561/1,542 | 1,596/1,714 | 0.29 | 0.30 | 0.27 |
| rs7041 | 4 | 72,837,198 | GC | T | G | 560/1,541 | 1,590/1,714 | 0.43 | 0.44 | 0.43 |
| rs10741657^e^ | 11 | 14,871,454 | CYP2R1 | G | A | 550/1,503 | 950/1,052 | 0.63 | 0.60 | 0.62 |
| rs3829251 | 11 | 70,872,207 | NADSYN1/  DHCR7 | A | G | 561/1,546 | -- | 0.15 | -- | 0.16 |
| rs6013897 | 20 | 52,175,886 | CYP24A1 | A | T | 559/1,543 | -- | 0.21 | -- | 0.21 |
| ^a^NCBI Build 36 | | | | | | | | | | |
| ^b^Among white controls | | | | | | | | | | |
| ^c^RAF obtained from the Database for Short Genetic Variations ([www.ncbi.nlm.nih.gov/snp](http://www.ncbi.nlm.nih.gov/snp)) | | | | | | | | | | |
| ^d^Among the subset of New England Case-Control study participants in which rs2282679 (position 72,827,247) was used as a proxy for rs4588 (*r*^2^=0.95), RAF=0.28 | | | | | | | | | | |
| ^e^rs2060793 (position 14,871,886) was substituted for rs10741657 (*r^2^*=0.88) in the New England Case-Control study participants genotyped on the iCOGS array ([49](#_ENREF_49)) | | | | | | | | | | |
